# Immunomodulatory Effects of a Prebiotic Formula with 2′-Fucosyllactose and Galacto- and Fructo-Oligosaccharides on Cyclophosphamide (CTX)-Induced Immunosuppressed BALB/c Mice via the Gut–Immune Axis

**DOI:** 10.3390/nu16203552

**Published:** 2024-10-19

**Authors:** Wanyun Ye, Hanxu Shi, Wentao Qian, Liping Meng, Meihua Wang, Yalin Zhou, Zhang Wen, Muke Han, Yile Peng, Hongliang Li, Yajun Xu

**Affiliations:** 1 Department of Nutrition and Food Hygiene, School of Public Health, Peking University, No. 38 Xueyuan Road, Beijing 100083, China; yewanyun_vera@bjmu.edu.cn (W.Y.); shihanxu@pku.edu.cn (H.S.); zylyingyang@163.com (Y.Z.); 1710306240@pku.edu.cn (Z.W.); hanmuke33@163.com (M.H.); 1810306134@pku.edu.cn (Y.P.); 2 Mengniu Hi-Tech Dairy Products (Beijing) Co., Ltd., Beijing 101100, China; qianwentao2006@163.com (W.Q.); menglipin@mengniu.cn (L.M.); 3 Inner Mongolia Mengniu Dairy (Group) Co., Ltd., Hohhot 011500, China; wangmeihua@mengniu.cn; 4 Beijing Key Laboratory of Toxicological Research and Risk Assessment for Food Safety, Peking University, No. 38 Xueyuan Road, Beijing 100083, China

**Keywords:** prebiotic formula, cyclophosphamide (CTX), immunosuppression, gut microbiota

## Abstract

**Obejectives:** This study explored the immunomodulatory effects of a prebiotic formula consisting of 2′-fucosyllactose (2′-FL), galacto-oligosaccharides (GOSs), and fructo-oligosaccharides (FOSs) (hereinafter referred to as 2FGF) in cyclophosphamide (CTX)-induced immunosuppressed BALB/c mice and its underlying mechanisms. **Methods:** Sixty healthy female BALB/c mice were randomly divided into the following groups: normal control (NC) group; CTX treatment (CTX) group; 2FGF low-dose (2FGF-L) group; 2FGF medium-dose (2FGF-M) group; and 2FGF high-dose (2FGF-H) group. An immunosuppressed model was established in the 2FGF-H group by intraperitoneal injection of 80 mg/kg CTX. After 30 days of 2FGF intervention, peripheral blood, spleen tissue, thymus tissue, and intestinal tissue from the mice were collected and analyzed. The changes in weight and food intake of the mice were recorded weekly. Hematoxylin-eosin (HE) staining was used to observe the histological change of the spleen tissue. Enzyme-linked immunosorbent assay (ELISA) was employed to detect cytokine levels in peripheral blood. Flow cytometry was used to analyze T lymphocyte subgroup ratio of splenic lymphocytes. Western blot analysis was conducted on intestinal tissues to assess the expression of proteins involved in the tight junction, toll-like receptor 4 (TLR4), mitogen-activated protein kinase (MAPK), and nuclear factor kappa-light-chain-enhancer of activated B cell (NF-κB) signaling pathways. Additionally, molecular techniques were used to analyze the intestinal microbiota. **Results:** The results showed that 2FGF restored CTX-induced splenic injury, increased the number of splenic T lymphocytes, and elevated serum cytokines such as interleukin-4 (IL-4) and IL-10. In the intestine, 2FGF upregulated the expression of intestinal epithelial tight junction proteins such as Claudin-1 and zonula occludens 1 (ZO-1), thereby enhancing intestinal barrier function and activating the MAPK and NF-κB pathways via TLR4. Furthermore, 2FGF elevated the α-diversity (Shannon and Simpson indices) of the gut microbiota in CTX-induced immunosuppressed mice, enriching bacteria species positively correlated with anti-inflammatory cytokines (e.g., IL-4) such as *g_Streptomyces* and *g_Bacillus* and negatively correlated with pro-inflammatory cytokines (e.g., IL-1β) such as *g_Saccharomyces*. The results suggest that 2FGF may enhance immunity via the gut–immune axis. **Conclusions:** The 2FGF prebiotic formula showed an immunomodulatory effect in CTX-induced immunosuppressed mice, and the mechanism of which might involve optimizing the gut flora, enhancing intestinal homeostasis, strengthening the intestinal barrier, and promoting the expression of immune factors by regulating the TLR-4/MAPK/NF-κB pathway.

## 1. Introduction

The gut is considered the largest peripheral lymphoid organ in the human body, containing 60% to 70% of the body’s peripheral immune cells [1]. The gastrointestinal tract is continually exposed to various dietary components, exogenous antigens, gut microbiota, and their respective metabolites. The gut barrier is essential to avoid the uncontrolled entry of harmful intestinal substances into the circulation. The gut microbiota, with bacterial gene richness that exceeds human genomic content by a factor of 100, plays an indispensable role in both enhancing and modulating the host’s immune response [2]. Disturbances in the gut microbiota and intestinal barrier integrity can rapidly lead to a series of immune responses, including gastrointestinal infections, inflammation, and allergies.

Galacto-oligosaccharides (GOSs) and fructo-oligosaccharides (FOSs), also known as prebiotic oligosaccharides (PBOs), are widely used in infant formula. Compared to infants fed regular infant formula, infant formula supplemented with GOSs/FOSs is similar to breast milk in preventing diarrhea, constipation, and respiratory infections in the first year after birth [3]. In recent years, the prebiotic efficacy of human milk oligosaccharides (HMOs) has received worldwide attention. 2′-fucosyllactose (2′-FL) is the most abundant HMO in breast milk, which can selectively stimulate the growth of beneficial bacteria in the gut, such as *Bifidobacterium*, increasing the concentration of short-chain fatty acids in the gut and upregulating the expression of intestinal tight junction proteins [4]. In in vitro human gut microbial ecosystems, 2′-FL has shown the ability to alter gut microbiota composition and regulate metabolites [4].

Prebiotic formulas containing 2′-FL, GOSs, and FOSs (hereinafter referred to as 2FGF) have been investigated in previous studies. Azagra et al. assessed the ameliorative effects of the 2′-FL+GOS/FOS combination on a rotavirus-induced diarrhea rat model and explored their impact on intestinal barrier function and systemic immune responses [5]. Combining these three oligosaccharides demonstrates a synergistic effect, where GOSs/FOSs exhibit the highest blocking effect, while 2′-FL serves as a significant promoter of the intestinal barrier. In certain variables, this combination also shows an additive effect. Another study evaluated the effect on the immune response of the murine influenza vaccination model using the 2FGF combination, which showed that this prebiotic combination increased serum immunoglobulin G1 (IgG1) and IgG2a levels and improved the influenza vaccine-specific T-helper 1 response and B cell activation in mesenteric lymph nodes [6]. These above studies provide findings on the effects of 2FGF on the gut and immunity. In this study, a cyclophosphamide (CTX)-induced immunosuppressed BALB/c mice model was established to investigate the immunomodulatory effects of different doses of 2FGF prebiotic formula and explore the potential mechanism through the gut–immune axis.

## 2. Materials and Methods

### 2.1. Materials and Reagents

2FGF (2′-FL/GOSs/FOSs = 1:3:3) (Inner Mongolia Mengniu Dairy (Group) Co., Ltd., Inner Mongolia, China), cyclophosphamide (CTX; analytical pure) (Sigma-Aldrich, St. Louis, MO, USA), sodium carboxymethyl cellulose (CMC-Na) and enzyme-linked immunosorbent assay (ELISA) kits for cytokines (DOp Biotechnology Co., Ltd., Beijing, China), multiplex cytokine detection kits (Millipore, Bedford MA, USA), 4% paraformaldehyde solution (Beijing Liangdong Technology Co., Ltd., Beijing, China), red blood cell lysis buffer (Beijing Dako Biotech Co., Ltd., Beijing, China), hematoxylin–eosin (HE) staining solution and phosphate-buffered saline (pBS) (Beijing pulizhicheng Biotechnology Co., Ltd., Beijing, China), claudin-1, zonula occludens 1(ZO-1), and occludin antibodies (Abcam Ltd., Cambridge, UK), Toll-like receptor 4 (TLR4), phosphorylated p38 (*p*-p38) and p38, phosphorylated ERK (*p*-ERK) and ERK, phosphorylated JNK (*p*-JNK) and JNK, and phosphorylated p65 (*p*-p65) and p65 (Cell Signaling Technology, Inc., Boston, MA, USA).

### 2.2. Animal Experiment Design

All experimental procedures were approved by the ethics committee of Peking University, China (permission number: LA2021107; approval date: 26 February 2021). Sixty specific pathogen-free (SPF) female BALB/c mice weighing 18–22 g (6 weeks old) were provided by the Department of Laboratory Animal Science of Peking University (Beijing, China, SCXK-2021-0013). The animals were housed, 4 mice per cage, in an SPF environment with a temperature of 20–24 °C, a humidity of 40–60%, and a 12 h light/dark cycle. After 3 days of adaptive feeding, the animals were randomly divided into the following 5 groups (12 animals per group): normal control (NC) group; CTX treatment (CTX) group; 2FGF low-dose (2FGF-L) group; 2FGF medium-dose (2FGF-M) group; and 2FGF high-dose (2FGF-H) group. The CTX-induced immunosuppressed BALB/c mice model (including CTX, 2FGF-L, 2FGF-M, and 2FGF-H groups) were given CTX (80 mg/kg bw) via intraperitoneal injection for three consecutive days, and the NC group was given an equal amount of normal saline via intraperitoneal injection [7]. After modeling, the low-, medium-, and high-dose 2FGF groups were administered 2FGF intubation intervention at dosages of 1, 2, and 4 g/kg body weight, respectively, for 30 consecutive days (equivalent to 5, 10, and 20 times the recommended intake for humans, respectively), while the NC and CTX groups were given an equal amount of distilled water via intubation. After the last administration, animals were fasted overnight for 12 h without water restriction, and peripheral blood was collected by retro-orbital blood collection. After euthanizing mice by cervical dislocation, samples including intestinal tissue, intestinal contents, thymus, and spleen were collected for relevant index detection.

### 2.3. Determination of the Body Weight, Thymus, and Spleen Indices

After 3 days of adaptive feeding, the body weight of the mice was recorded as the weight of Week 1 and then recorded every 7 days. After sacrifice, the thymus and spleen were collected and rinsed with normal saline, and the liquid was absorbed with filter paper before weighing. Thymus (or spleen) index = thymus (or spleen) weight/body weight (mg/g).

### 2.4. Histological Analysis of the Spleen

Part of the mice’s spleen was fixed in 4% paraformaldehyde, paraffin-embedded, cut into 3 μm-thick sections, and stained with hematoxylin–eosin (HE). The histological changes in the spleen were observed under a fluorescence microscope (OLYMPUS BX43, Tokyo, Japan).

### 2.5. Flow Cytometry Analysis of the Spleen

The T lymphocyte subgroup ratio of splenic lymphocytes was analyzed with a flow cytometer (Calibur; Becton Dickinson, Franklin Lakes, NJ, USA) [8]. The mouse spleen was harvested to prepare a single-cell suspension and centrifuged at 300× *g* for 5 min, followed by discarding the supernatant and resuspending the cell precipitate with 2 mL of 1 × lysis buffer. After 5 min of ice incubation, the cells were treated with 2 mL of PBS, recentrifuged, and resuspended to a concentration of 1 × 10^6^ cells/100 μL. In flow tubes, 100 μL of this suspension was combined with FITC-conjugated CD3 antibody, PE-conjugated CD4 antibody, and APC-conjugated CD8 antibody and then incubated in the dark for 30 min with intermittent mixing. Post-wash with PBS and centrifugation, the cells were fixed in 300 μL of 2% paraformaldehyde for the flow cytometric analysis of the CD3^+^, CD4^+^, and CD8^+^ cell percentages.

### 2.6. Determination of Cytokines

The serum levels of interleukin-1β (IL-1β), IL-2, IL-6, IL-22, IL-4, and IL-10 were determined using mouse enzyme-linked immunosorbent assay (ELISA) kits (DOP Biotechnology Co., Ltd., Beijing, China) according to the manufacturer’s instructions.

### 2.7. Western Blot Analysis

Western blot analysis was conducted to detect the expression of tight junction protein and TLR4, mitogen-activated protein kinase (MAPK), and nuclear factor kappa-light-chain-enhancer of activated B cell (NF-κB) pathway-related proteins in intestinal tissues following a previously reported protocol [9].

The samples were incubated with primary antibodies, including Claudin-1 (ab307692), ZO-1 (ab276131), TLR4 (ab209217), and β-actin (ab8227) from Abcam Ltd. (Cambridge, UK) and p38 (8690), *p*-p38 (4511), ERK1/2 (4695), *p*-ERK1/2 (4370), JNK (9252), *p*-JNK (4668), p65 (8242), and *p*-p65 (3033) from Cell Signaling Technology (Boston, MA, USA). The secondary antibody from Abcam Ltd. (Cambridge, UK) was conjugated with horseradish peroxidase for 2 h. The band density of each protein was quantified using ImageJ software (version 1.8.0).

### 2.8. Metagenomic Analysis of Gut Microbiota

Fresh mice intestinal contents of the ascending colon were collected and frozen at −80 °C for later analysis. DNA was extracted using a modified QIAamp Fast DNA Stool Mini Kit (Qiagen, Hilden Germany), involving homogenization with InhibitEX buffer and glass beads, followed by bead beating with a FASTPREP-24 (Aosheng Biotech, Anyang, China) [10]. DNA was purified according to the manufacturer’s instructions; its concentration was measured with NanoDrop (Thermo Scientific, Waltham, MA, USA) and Qubit 2.0 (Invitrogen, Carlsbad, CA, USA), and its quality was assessed using agarose gel electrophoresis. DNA libraries with approximately 400 bp insert sizes were constructed using a VAHTS Universal Plus DNA Library Prep Kit for Illumina (Vazyme Biotech Co., Ltd., Nanjing, China). The library sizes were evaluated with an Agilent 2100 bioanalyzer (Agilent Technologies, Wokingham, UK) and an Agilent 2100 DNA 1000 Kit. The samples underwent 150 bp paired-end sequencing on a Novaseq X Plus platform (Illumina, Inc., San Diego, CA, USA). Illumina raw reads were filtered for adaptor contamination, more than three ambiguous N bases, low-quality reads (Q < 20), and less than 60% high-quality bases (Phred score ≥ 20). Clean reads were aligned to bacterial genomes from GenBank using SOAPaligner (version 2.21), with reads mapping to the host genome discarded. The remaining reads were aligned to the NCBI database for the detection of known bacteria, fungi, viruses, and archaea using SOAPaligner 2.21. The aligned reads were classified at various taxonomic levels (Kingdom to Species) to determine classification and abundance, generating a taxonomic relative abundance profile. In order to assess the effects of 2FGF on distinct gut microbiota within immunosuppressed mice, a selection of strains exhibiting comparatively higher abundance was screened. PCoA analysis was performed using the ade4 package in R (version 4.4.2). The effect size of differentially abundant genes across various samples was determined using the linear discriminant analysis effect size (LEfSe).

### 2.9. Statistical Analysis

All statistical evaluations were performed using SPSS software (version 25.0 SPSS Inc.; Chicago, IL, USA), while graphs were created using Origin (version 2024b, OriginLab Corporation; Northampton, MA, USA). Each result was tested in triplicate, which means that each sample or set of data in the experiment is tested three times to ensure the reliability of the results, and presented as means ± standard deviations. Differences between groups were assessed using a one-way ANOVA, followed by Fisher’s LSD test. The Shannon and Simpson indices were used to examine the evenness and variety of microbiota, using the Bray–Curtis distance to obtain the distance matrix among samples, followed by principal coordinates analysis (PCoA) to assess the β-diversity of the microbial community. Linear discriminant analysis effect size (LEfSe) analysis was conducted to identify significant microbial taxa. Spearman’s correlation analysis was carried out to clarify the connection between the gut microbiota and cytokines. *p* < 0.05 was considered statistically significant.

## 3. Results

### 3.1. Effect of 2FGF on CTX-Induced Immunosuppressed Mice

#### 3.1.1. Effect of 2FGF on Body Weight, Food Intake, Immune Organ Indices, Histological Changes, and T Lymphocyte Subgroups

**Figure 1:**
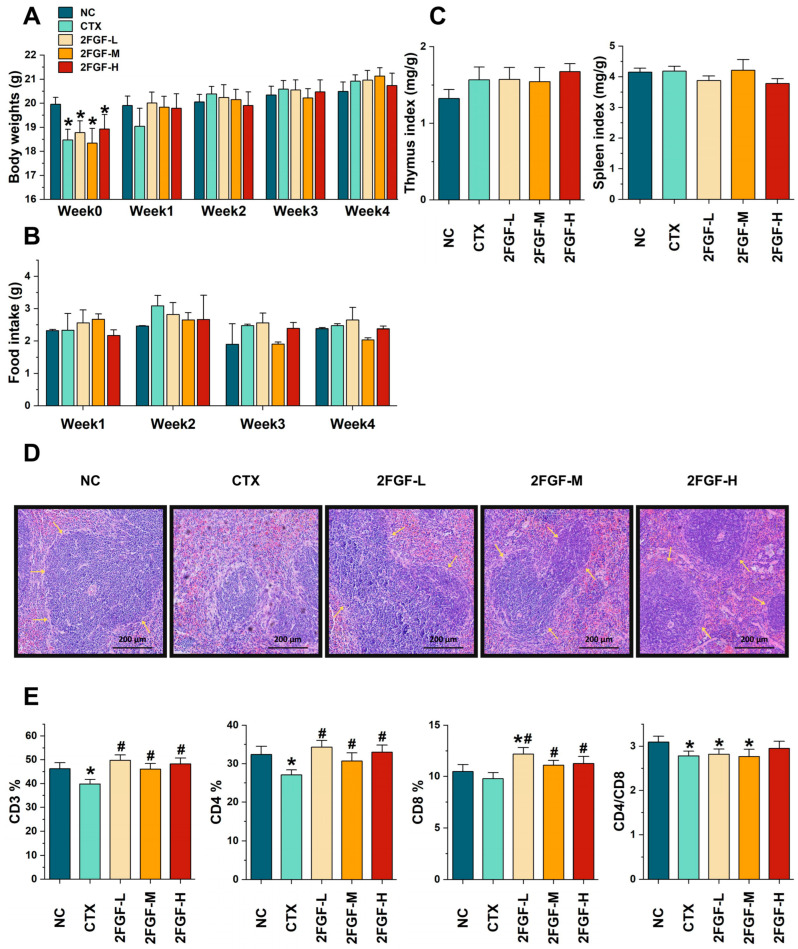
The effect of 2FGF on body weight (**A**), food intake (**B**), immune organ indices (**C**), histological observation of the spleen (original magnification: ×40) (**D**), and splenic T lymphocyte subgroups in CTX-induced immunosuppressed mice (**E**). NC, normal control group; CTX, CTX-induced immunosuppressed model group; 2FGF-L, 2FGF (2′-FL/GOS/FOS = 1:3:3) administered at 1 g/kg bw; 2FGF-M, 2FGF administered at 2 g/kg bw; 2FGF-H, 2FGF administered at 4 g/kg bw. The yellow arrows in (**D**) indicate the boundaries of the red and white pulp of the spleen. Data are presented as means ± standard deviations (n = 12). Significant differences compared to the NC group are indicated by * (*p* < 0.05), and significant differences compared to the CTX group are indicated by # (*p* < 0.05).

Intraperitoneal injection of CTX significantly reduced the body weight of the mice after modeling (Figure 1A), and there were no statistical differences between any group during the subsequent period. There were no significant differences in food intake, thymus index, or spleen index among the groups (Figure 1B,C). The histological analysis of the spleen is shown in Figure 1D, where the NC group showed a normal splenic tissue structure with clear boundaries between the white and red pulp and well-developed splenic nodules. The CTX group had blurred boundaries between the white and red pulp, with atrophy of the white pulp area. After treatment with 2FGF, a significant improvement in the histological morphology of the spleen was observed, with clear boundaries between the white and red pulp and an increase in the white pulp area. The results of the splenic T lymphocyte subgroups are shown in Figure 1E; compared to the NC group, the percentages of CD3^+^ and CD4^+^ T lymphocytes and the CD4^+^/CD8^+^ ratio in the CTX group were significantly reduced (*p* < 0.05). Compared to the CTX group, the percentages of CD3^+^, CD4^+^, and CD8^+^ T lymphocytes after intervention with 2FGF were significantly increased (*p* < 0.05), and the CD4^+^/CD8^+^ ratio in the 2FGF-H group showed an increasing trend with no statistical significance (*p* > 0.05).

#### 3.1.2. Effect of 2FGF on Serum Cytokine Levels

The cytokine results are shown in Table 1. Compared to the NC group, the serum levels of pro-inflammatory cytokines such as IL-2 and IL-6 showed an increasing trend in the CTX group, while anti-inflammatory cytokine IL-10 was decreased (*p* > 0.05). Compared to the CTX group, the serum levels of anti-inflammatory cytokines, including IL-4 and IL-10, were significantly increased in the 2FGF-H group (*p* < 0.05), suggesting that 2FGF may reverse immune disorders caused by CTX.

### 3.2. Effect of 2FGF on the Intestines of CTX-Induced Immunosuppressed Mice

#### 3.2.1. Effect of 2FGF on Intestinal Epithelial Barrier Functions

**Figure 2:**
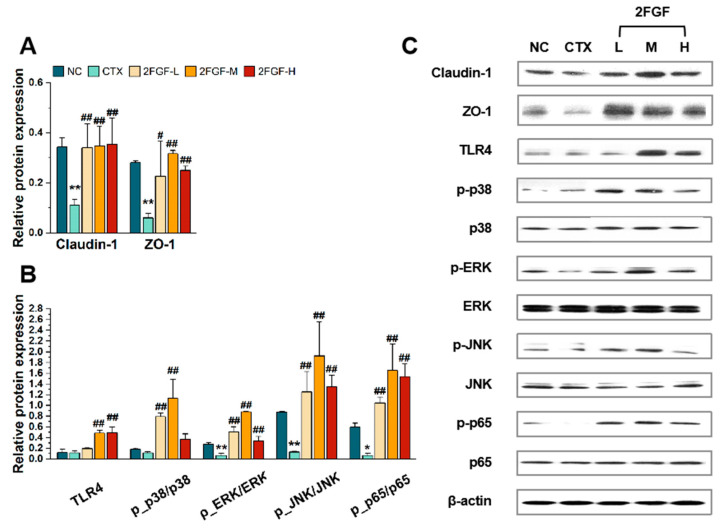
The effect of 2FGF on the protein expression of Claudin-1 and ZO-1 (**A**), TLR-4, *p*-p38, p38, *p*-ERK, ERK, *p*-JNK, JNK, *p*-p65, and p65 in the large intestines (ascending colon) of CTX-induced immunosuppressed mice (**B**), with representative Western blot images (**C**). Data are presented as means ± standard deviations (n = 3). Significant differences compared to the NC group are indicated by * (*p* < 0.05) and ** (*p* < 0.01), and significant differences compared to the CTX group are indicated by # (*p* < 0.05) and ## (*p* < 0.01).

Western blot was used to detect the expression of intestinal tight junction proteins, including Claudin-1 and ZO-1 proteins, in the mice’s intestinal epithelium. Compared to the NC group, the expression of intestinal tight junction proteins was significantly reduced after CTX treatment (Figure 2A). However, under 2FGF intervention, the expression of Claudin-1 and ZO-1 significantly increased (*p* < 0.01). These results indicate that 2FGF has a protective effect on the intestinal barrier.

#### 3.2.2. Effect of 2FGF on the TLR4/MAPK/NF-κB Pathway

As shown in Figure 2B, CTX inhibited the MAPK and NF-κB pathways and significantly suppressed the expression of phosphorylated ERK, JNK, and p65 (*p* < 0.05). 2FGF played an important role in reversing the inhibition of the MAPK and NF-κB pathways. Compared to the CTX group, the expression of TLR4 protein significantly increased in the 2FGF-M and 2FGF-H groups (*p* < 0.01). The ratio of *p*-p38/p38 significantly increased in the 2FGF-L and 2FGF-M groups (*p* < 0.01). The ratios of *p*-ERK/ERK, *p*-JNK/JNK, and *p*-p65/p65 significantly increased in all three dose groups of 2FGF (*p* < 0.01). 2FGF can activate the NF-κB and MAPK signaling pathways in the intestines of CTX-induced immunosuppressed mice.

#### 3.2.3. Effect of 2FGF on Gut Microbiota

**Figure 3:**
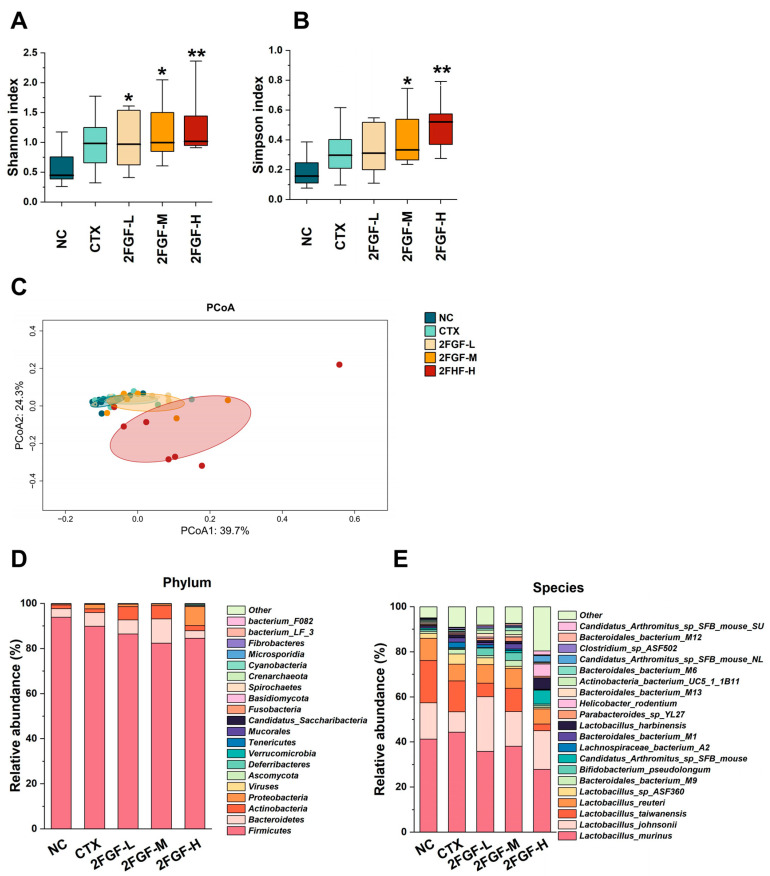
The effect of 2FGF on the Shannon index (**A**), Simpson index (**B**), PCoA analysis (**C**), phylum-level relative abundance (**D**), and species-level relative abundance (**E**) of the gut microbiota in CTX-induced immunosuppressed mice. Data are presented as means ± standard deviations (n = 8). Significant differences compared to the NC group are indicated by * (*p* < 0.05) and ** (*p* < 0.01).

Metagenomic methods were employed to analyze the gut microbiota composition across mice groups, utilizing the Shannon and Simpson indices to assess α-diversity, as depicted in Figure 3A,B. The Shannon and Simpson indices of the CTX group were not significantly different from the NC group. However, 2FGF intervention significantly enhanced the Shannon index in all three dose groups (Figure 3A) and the Simpson index in the 2FGF-M and 2FGF-H groups (Figure 3B), indicating an improvement in microbiota richness and evenness. β-diversity was evaluated using Bray–Curtis’s principal coordinate analysis (PCoA), as shown in Figure 3C, where the 2FGF-H group was significantly separated from the other groups on axis 1.

To further study the effect of 2FGF on the abundance of specific gut microbiota, we compared the composition of gut microbiota at the phylum and species levels among the groups. As shown in Figure 3D, at the phylum level, the main microbial communities of the mice in each group were *Firmicutes*, *Bacteroidetes*, *Actinobacteria*, and *Proteobacteria*. Compared to the NC group, the relative abundance of *Proteobacteria* in the CTX group significantly increased. Treatment with 2FGF at low and medium doses increased the relative abundance of *Actinobacteria* and decreased the relative abundance of *Proteobacteria*. The relative abundances of *Actinobacteria* and *Proteobacteria* showed the opposite change after treatment with a high dose of 2FGF. At the species level, *Lactobacillus_johnsonii* was notably diminished in the CTX group but was restored by 2FGF supplementation. This beneficial bacterium is known to preserve intestinal barrier integrity, modulate gut microbiota composition, elevate short-chain fatty acid levels, and mitigate intestinal inflammation in mice [11].

### 3.3. Correlation Analysis of Differential Microbiota and Serum Inflammatory Markers

**Figure 4:**
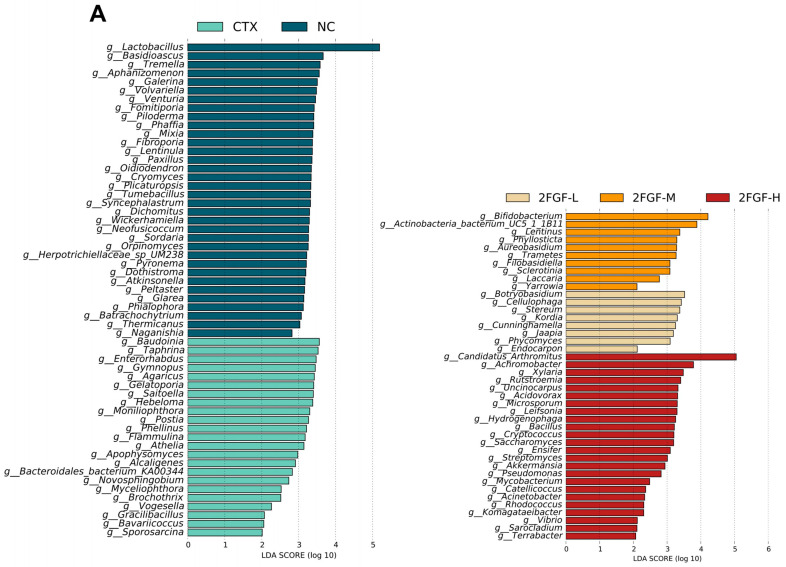

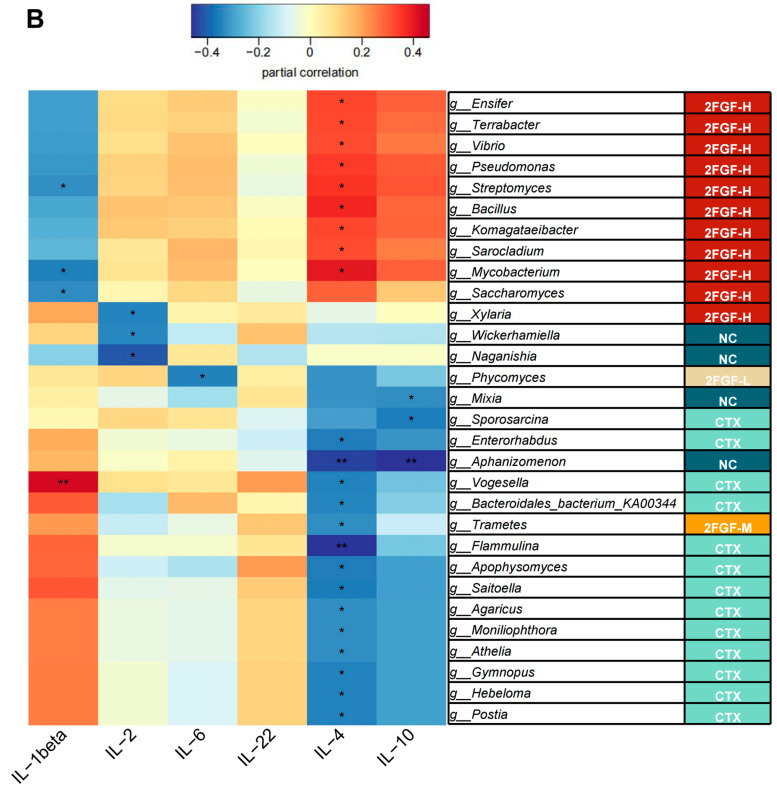
Histogram of LDA scores (**A**). Correlation analysis between differential genera and cytokines (**B**). Red indicates a positive correlation, and blue indicates a negative correlation. Significant differences are indicated by * (*p* < 0.05) and ** (*p* < 0.01).

According to the LDA score distribution map, there were 34, 22, 8, 10, and 24 differential species in the NC, CTX, 2FGF-L, 2FG-M, and 2FGF-H groups, respectively (Figure 4A). By analyzing the correlation between the relative abundance of enriched gut microbiota and cytokine levels in each group, we further explored the potential mechanism by which 2FGF improves CTX-induced immunosuppression. Figure 4B is a heat map of the correlation between cytokines and the flora with significant differences between groups, and each differential flora is followed by the group in which they are enriched. In general, the bacteria exhibiting significant intergroup disparities were predominantly enriched within the CTX and 2FGF-H groups. As depicted in Figure 4B, the bacteria enriched in the CTX group negatively correlated with IL-4 and IL-10 levels, whereas those enriched in the 2FGF-H group positively correlated with these cytokine levels.

The genera enriched in the NC group, *g_Wickerhamiella* and *g_Naganishia*, were significantly negatively correlated with serum IL-2 levels, while *g_Mixia* and *g_Aphanizomenon* were significantly negatively correlated with IL-4 and IL-10 levels. The genera enriched in the CTX group, *g_Sporosarcina*, *g_Enterorhabdus*, *g_Aphanizomenon*, *g_Vogesella*, *g_Bacteroidales_bacterium_ KA00344*, *g_Flammulina*, *g_Apophysomyces*, *g_Saitoella*, *g_Agaricus*, *g_Moniliophthora*, *g_Athelia*, *g_Gymnopus*, *g_Hebeloma*, and *g_Postia*, were significantly negatively correlated with IL-4 levels. The genus enriched in the 2FGF-L group, *g_Phycomyces*, was significantly negatively correlated with IL-6 levels. The genus enriched in the 2FGF-M group, *g_Trametes*, was significantly negatively correlated with IL-4 levels. The genera enriched in the 2FGF-H group, *g_Ensifer*, *g_Terrabacter*, *g_Vibrio*, *g_Pseudomonas*, *g_Streptomyces*, *g_Bacillus*, *g_Komagataeibacter*, *g_Sarocladium*, and *g_Mycobacterium*, were significantly positively correlated with IL-4 levels, while *g_Streptomyces*, *g_Mycobacterium*, and *g_Saccharomyces* were significantly negatively correlated with IL-1β levels and *g_Xylaria* was significantly negatively correlated with IL-2 levels. These results suggest that 2FGF supplementation changes the composition of the gut microbiota and affects the concentration of serum cytokines.

## 4. Discussion

In this study, we observed that a 30-day 2FGF intervention mitigated CTX-induced splenic injury, elevated the percentage of splenic CD3^+^, CD4^+^, and CD8^+^ T lymphocytes, protected the intestinal barrier, and improved the gut microbiota. A high-dose intraperitoneal injection of CTX over a short period is a common method for establishing an immunosuppressed mouse model. CTX destroys the structure of DNA, induces apoptosis in immune cells, impedes lymphocyte proliferation and differentiation, and diminishes lymphocyte counts, thereby dampening both cellular and humoral immune responses [12]. The spleen, consisting of lymphocyte-rich white pulp, a marginal zone, and macrophage-abundant red pulp, exhibited blurred demarcations between the white and red pulp in CTX-treated mice, with a concomitant reduction in white pulp area. Treatment with 2FGF ameliorated splenic damage. Previous studies have found that a conjugate of GOSs and whey protein isolate can repair the damaged boundary between red and white pulp [13]. The immune function of lymphocytes is modulated by distinct cell surface markers. The main surface marker of T cells is CD3. In the adaptive immune response, CD4^+^ and CD8^+^ T lymphocytes function as principal helper and cytotoxic T cells, respectively [14]. 2FGF intervention reversed the reduction in CD3^+^, CD4^+^, and CD8^+^ T cells caused by CTX, with CD4^+^ T cells, as helper T cells, exerting regulatory roles in immune function through diverse mechanisms. They can activate immune responses, secrete cytokines, promote the activation of B cells, and activate the cytotoxic function of CD8^+^ T cells [15]. Prior research has found that treatment with GOSs can significantly increase the CD4^+^/CD8^+^ ratio in CTX-induced immunosuppressed mice [16], aligning with the trends observed in our study.

Cytokine IL-4 is a key cytokine that promotes the differentiation and function of helper T cells type 2 (TH2) and, when combined with other cytokines such as IL-5, IL-9, and IL-13, can drive the proliferation of B cells and the class switching of immunoglobulins to immunoglobulin E (IgE), thereby enhancing humoral immunity [17]. IL-10, an anti-inflammatory cytokine, is ubiquitously expressed by immune cells and is integral to immune response modulation during infections [18]. In this study, IL-4 and IL-10 levels significantly increased in the high-dose 2FGF treatment group, which is consistent with previous findings. Zhao et al. reported that intervention with a combination of *Bacillus coagulans* 13002 and FOSs in CTX-induced immunosuppressed mice elevated intestinal mucosal IL-4 levels [19]. Tang et al. reported a significant increase in serum IL-4 levels due to GOS treatment in CTX-induced immunosuppressed mice [16]. 2′-FL intervention was found to enhance IL-10 mRNA expression and substantially elevate IL-10 serum concentrations in CTX-treated mice [20].

TLR4, a pivotal pattern recognition receptor within the innate immune system, is instrumental in identifying pathogen-associated molecular patterns and initiating the downstream MAPK and NF-κB signaling pathways [21]. In this study, the upregulation of TLR4 expression post-2FGF treatment suggests its potential role in modulating the activation of these signaling pathways. MAPK, a conserved family of protein kinases, is integral to cellular signal transduction, encompassing the p38, ERK, and JNK subfamilies. Phosphorylation of the above three proteins is indicative of MAPK pathway activation [22]. NF-κB is a critical transcription factor family, and phosphorylation of p65 is a key step in the activation of the NF-κB signaling pathway, which in turn induces the expression of inflammatory mediators such as cytokines, chemokines, and adhesion factors [23]. The intervention of 2FGF reversed the inhibitory effect of CTX on MAPK and NF-κB signaling pathways, potentially modulating the inflammatory response through these pathways. This study found that CTX significantly inhibited the phosphorylation of ERK, JNK, and p65, while 2FGF intervention notably enhanced the phosphorylation of p38, ERK, JNK, and p65. Previous studies have demonstrated that treatment with GOSs can significantly increase the phosphorylation of p65, ERK, JNK, and p38 in the peritoneal macrophages of CTX-treated mice [16]. The activation of p38 phosphorylation was also found to be related to the promotion of IL-10 production, aligning with the elevated IL-10 levels observed in our study [24]. However, in some studies, the activation of the MAPK and NF-κB pathways appears to be associated with elevated levels of cytokines such as IL-1β and IL-6, which were not found to be statistically different between the various groups in this study. This may be related to the compositional components of 2FGF involved in the regulation of other signaling pathways. 2′-FL has been found to reduce the overexpression of IL-1α, IL-1β, IL-6, and IL-8 induced by particulate matter (PM)_10_ by modulating the activation of the phosphatidylinositol 3-kinase (PI3K)/Akt pathway [25]. A mixture of 2′-FL with GOSs and FOSs was found in previous studies to significantly increase the levels of butyrate and propionate in the gut. Short-chain fatty acids (SCFAs) are widely believed to reduce the levels of pro-inflammatory factors, which may also mask the pro-inflammatory effects of the MAPK pathway in this study [6]. In this study, the concentrations of intestinal SCFAs were not assessed. To elucidate the mechanism of how 2FGF modulates cytokine expression, it is imperative in future investigations to include an assessment of SCFA levels.

Intestinal barrier disruption and functional impairment can escalate intestinal permeability, permitting the translocation of pathogens and noxious substances such as endotoxins, leading to tissue injury and systemic inflammation [26]. Tight junction protein expression serves as a robust biomarker for intestinal barrier integrity assessment. Our study identified a reduction in Claudin-1 and ZO-1 expression in CTX-induced immunosuppressed mice, with 2FGF intervention significantly ameliorating the compromised intestinal barrier. CTX-induced intestinal epithelial damage has been validated in previous study [27]. Liu et al. utilized 2′-FL and GOSs in DSS-induced colitis mice, revealing that both interventions markedly improved intestinal barrier damage, effectively upregulating ZO-1 and occludin expression in the colon, with 2′-FL demonstrating superior efficacy over GOSs [28]. Another study reported that FOSs could alleviate the loss of ZO-1, occludin, and Claudin-1 in the colonic epithelium induced by DSS [29].

**Figure 5:**
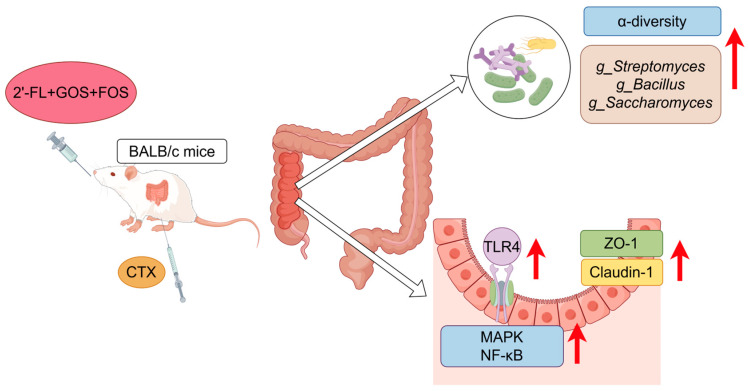
Possible immunomodulatory mechanism of the 2FGF prebiotic formula in CTX-induced immunosuppressed mice.

Gut microbiota are crucial for intestinal barrier development, pathogen resistance, and immune system maturation. In this study, the CTX group exhibited a significant increase in the relative abundance of *Proteobacteria* and a decrease in *Lactobacillus_johnsonii*. *Lactobacillus johnsonii* is recognized for its role in maintaining the integrity of the intestinal barrier, adjusting the balance of gut bacteria, increasing the levels of short-chain fatty acids, and reducing intestinal inflammation in mice, as reported in reference [11]. Treatment with 2FGF reversed these alterations, significantly enhancing α-diversity, with a high dose inducing β-diversity differentiation from the CTX group. Zhong et al. reported similar findings with CTX treatment in mice, with increased *Proteobacteria* and decreased *Lactobacillaceae* [30]. *Proteobacteria*, which are significantly enriched in pro-inflammatory environments, are a source of inflammatory lipopolysaccharides (LPSs) [31]. *Lactobacillaceae*, as a representative probiotic, has been proven to reduce intestinal damage, bolster intestinal immune barriers, and repair epithelial cell barriers. Recent studies have reported that *Lactobacillaceae* can restore the altered immune cell ratio in inflammatory bowel disease (IBD), enhance anti-inflammatory ability, and inhibit pro-inflammatory ability [32]. 2′-FL, GOSs, and FOSs have all been found to increase gut microbiota diversity [33,34]. *g_Streptomyces*, known for its bioactive alkaloids, exhibits antibacterial, antitumor, and anti-inflammatory properties [35]. *Bacillus_coagulans_BACO-17*, a member of *g_Bacillus*, has been reported to significantly downregulate pro-inflammatory cytokines and dose-dependently inhibit the expression of IL-1β and IL-6 induced by TNF-α [36]. *Saccharomyces_boulardii (CNCM I-745)*, a *g_Saccharomyces* species, has been found to regulate gut microbiota, promote the expression of intestinal tight junction proteins, decrease intestinal permeability, and reduce pro-inflammatory cytokine serum levels in arthritic rats [37]. In our study, *g_Streptomyces* and *g_Bacillus* were enriched in the 2FGF-H group and showed a significant positive correlation with anti-inflammatory cytokine IL-4 levels, and *g_Saccharomyces* was enriched in the 2FGF-H group and showed a significant negative correlation with the pro-inflammatory cytokine IL-1β level. Therefore, 2FGF may regulate the body’s immune capacity by enriching gut flora positively correlated with anti-inflammatory cytokines. These bacteria species may restore intestinal homeostasis by elevating the expression of tight junction proteins, thereby providing more favorable conditions for the enhancement of gut microbiota diversity. The strengthening of the intestinal barrier effectively prevents the entry of pathogens and harmful substances into circulation. An increase in gut microbiota diversity may stimulate the secretion of SCFAs, leading to elevated levels of anti-inflammatory factors in the body.The immunomodulatory mechanism of 2FGF prebiotic formula on immunosuppressed mice is shown in Figure 5.

There are inherent limitations within the scope of this study. The research was conducted using a CTX-induced immunosuppressed mouse model and preliminarily explored the potential mechanisms by which 2FGF ameliorates immune function. The results indicated that 2FGF could enhance the relative abundance of beneficial bacteria, including *g_Streptomyces*, *g_Bacillus*, and *g_Saccharomyces*, that strengthen the intestinal barrier and activate the MAPK and NF-κB pathways, which may be relevant pathways to ameliorate immunosuppression. Further in vitro studies are warranted to continue exploring the specific mechanisms by which 2FGF enhances immune function. Concurrently, to translate the immunoenhancing potential of 2FGF into food applications, it is necessary to conduct clinical trials to validate its health effects in human.

## 5. Conclusions

The 2FGF prebiotic formula showed an immunomodulatory effect in CTX-induced immunosuppressed mice, and the mechanism of which might involve optimizing the gut flora, enhancing intestinal homeostasis, strengthening the intestinal barrier, and promoting the expression of immune factors by regulating the TLR-4/MAPK/NF-κB pathway. This combination has good prospects for use in infant formula to promote infant immunity.

## Figures and Tables

**Table 1:** The effect of 2FGF on peripheral blood cytokines in CTX-induced immunosuppressed mice (n = 12).

Group	IL-1β (pg/mL)	IL-2 (pg/mL)	IL-6 (pg/mL)	IL-22 (pg/mL)	IL-4 (pg/mL)	IL-10 (pg/mL)
NC	0.75 ± 0.41	0.93 ± 0.80	3.86 ± 2.18	6.76 ± 4.37	0.49 ± 0.07	1.04 ± 0.39
CTX	0.73 ± 0.39	1.33 ± 1.04	4.78 ± 2.70	5.70 ± 3.82	0.46 ± 0.06	0.75 ± 0.24
2FGF-L	0.76 ± 0.36	1.17 ± 0.88	3.11 ± 3.25	7.83 ± 2.85	0.49 ± 0.12	1.21 ± 0.97
2FGF-M	0.69 ± 0.34	1.09 ± 0.75	3.65 ± 1.44	7.00 ± 5.49	0.49 ± 0.04	1.48 ± 0.85 #
2FGF-H	0.82 ± 0.51	1.07 ± 1.05	4.37 ± 2.84	6.42 ± 3.64	0.55 ± 0.05 *#	1.40 ± 0.29 #

All data are presented as means ± standard deviations. Significant differences compared to the NC group are indicated by * (*p* < 0.05), and significant differences compared to the CTX group are indicated by # (*p* < 0.05).

## Data Availability

The datasets used during the current study are available from the corresponding author upon reasonable request.
